# Multiple Sense and Antisense Promoters Contribute to the Regulated Expression of the *isc-suf* Operon for Iron-Sulfur Cluster Assembly in *Rhodobacter*

**DOI:** 10.3390/microorganisms7120671

**Published:** 2019-12-10

**Authors:** Xin Nie, Bernhard Remes, Gabriele Klug

**Affiliations:** Institute of Microbiology and Molecular Biology, University of Giessen, Heinrich-Buff-Ring 26-32, D-35392 Giessen, Germany; Xin.Nie@bio.uni-giessen.de (X.N.); Bernhard.Remes@gmx.de (B.R.)

**Keywords:** iron-sulfur cluster, *isc* genes, *suf* genes, antisense promoters, OxyR, IscR, Irr

## Abstract

A multitude of biological functions relies on iron-sulfur clusters. The formation of photosynthetic complexes goes along with an additional demand for iron-sulfur clusters for bacteriochlorophyll synthesis and photosynthetic electron transport. However, photooxidative stress leads to the destruction of iron-sulfur clusters, and the released iron promotes the formation of further reactive oxygen species. A balanced regulation of iron-sulfur cluster synthesis is required to guarantee the supply of this cofactor, on the one hand, but also to limit stress, on the other hand. The phototrophic alpha-proteobacterium *Rhodobacter sphaeroides* harbors a large operon for iron-sulfur cluster assembly comprising the *iscRS* and *suf* genes. IscR (**i**ron-**s**ulfur **c**luster **r**egulator) is an iron-dependent regulator of *isc-suf* genes and other genes with a role in iron metabolism. We applied reporter gene fusions to identify promoters of the *isc-suf* operon and studied their activity alone or in combination under different conditions. Gel-retardation assays showed the binding of regulatory proteins to individual promoters. Our results demonstrated that several promoters in a sense and antisense direction influenced *isc-suf* expression and the binding of the IscR, Irr, and OxyR regulatory proteins to individual promoters. These findings demonstrated a complex regulatory network of several promoters and regulatory proteins that helped to adjust iron-sulfur cluster assembly to changing conditions in *Rhodobacter sphaeroides*.

## 1. Introduction

Proteins containing iron-sulfur (Fe-S) clusters are present in almost all living organisms. They have diverse and often essential functions, for example, electron carriers in redox reactions, in redox sensing, oxidative stress defense, biosynthesis of metal-containing cofactors, DNA replication and repair, regulation of gene expression, and tRNA modification. Fe-S clusters are believed to be among the first catalysts to have evolved [1,2]. With the appearance of oxygenic photosynthesis, increasing oxygen levels drastically decrease iron availability [3]. Besides molecular oxygen, different reactive oxygen species (ROS) appear, which are very harmful to living cells since they can damage proteins, lipids, and nucleic acids. Molecular oxygen and ROS also destabilize Fe-S clusters, leading to the release of Fe^2+^ ions that, in turn, potentiate oxygen toxicity by the production of hydroxyl radicals in the Fenton reaction [4]. As a consequence, organisms have to develop multicomponent systems that promote the biogenesis of Fe-S proteins while protecting the cellular surrounding from the deleterious effects of free iron.

Different systems for the assembly of Fe-S clusters into biological macromolecules have evolved: the Isc (**i**ron-**s**ulfur **c**luster) system was identified as the system for generalized Fe-S protein maturation in *Azotobacter vinelandii* [5] and other bacteria. In *Escherichia coli*, the *isc* operon encodes the regulator IscR (**i**ron-**s**ulfur **c**luster **r**egulator), a cysteine desulfurase (IscS), a scaffold protein (IscU), an A-type carrier protein (IscA), a DnaJ-like co-chaperone (HscB), a DnaK-like chaperone (HscA), and a ferredoxin (fdx) [6]. The Isc machinery is widely conserved from prokaryotes to higher eukaryotes. Later, another operon for Fe-S cluster biogenesis, *suf*, was discovered in *E. coli* [7]: the *suf* operon encodes an A-type protein (SufA), a heterodimeric cysteine desulfurase (SufS and SufE), and a pseudo-ABC transporter (SufB, SufC, and SufD) that could act as a scaffold. Components of the Suf system are also found in other bacteria, including cyanobacteria and chloroplasts. The number and type of *isc* and *suf* operons, as well as their composition, vary among bacterial species. In *E. coli*, most of the Fe-S cluster biogenesis under non-stress conditions is catalyzed by the housekeeping Isc pathway, while the Suf system functions primarily under oxidative stress and/or iron starvation [8,9,10,11,12].

Fe-S cluster biogenesis systems have to respond to environmental stimuli to maintain and repair the pool of Fe-S proteins under changing environmental conditions. The current understanding of the regulation of genes for Fe-S cluster assembly is mostly limited to the model organism *E. coli* and a few other members of the gamma-proteobacteria. The main regulator of Fe-S assembly in *E. coli* is IscR, but other regulatory proteins, such as Fur (an iron-dependent regulator) and OxyR (an oxidative stress-dependent regulator) also contribute to regulated *isc* and *suf* operon expression [9,10,12]. The first gene of the *iscRSUA-hscBA-fdx* operon encodes the DNA-binding IscR protein that can coordinate a [2Fe-2S] cluster, which is assembled by the Isc system [13,14]. In *E. coli*, IscR regulates at least 40 genes comprising the *isc* and *suf* operons, genes for other Fe-S containing proteins, and genes encoding surface structures (*fim* and *flu*) or of unknown functions [9]. Holo-IscR (IscR containing a Fe-S cluster) represses its gene and the rest of the *isc* operon. This repression is released under conditions unfavorable for Fe-S maturation of IscR [6]. Thus, IscR also functions as a sensor for Fe-S homeostasis. Apo-IscR (protein lacking the Fe-S cluster) activates *suf* operon expression [15] in *E. coli*. Induction of the *isc* and *suf* operons by apo-IscR occurs under iron-limiting conditions and oxidative stress [8,10,11]. A switch from the Isc to the Suf system when the iron is limiting is also promoted by the non-coding sRNA RhyB that is under negative control of Fur (**f**erric **u**ptake **r**egulator) [16]. RhyB base pairs with the Shine-Dalgarno sequence of *iscS*, leading to the degradation of the 3’ part of the *iscRSUA-hscBA-fdx* operon mRNA. The 5´ part of the polycistronic mRNA, which contains *iscR*, is stabilized and translated [17]. This favors the formation of apo-IscR, which consequently induces *suf* expression.

Regulation of the *isc* and *suf* operons by stress signals also involves the regulators Fur, OxyR, and RhyB. The iron-sensing regulator protein Fur binds to the *suf* promoter as Fur-Fe^2+^ represses the *suf* genes [10]. OxyR, a known sensor protein for oxidative stress in *E. coli* and many other bacteria, also acts as an activator of the *suf* operon [12]. It was also proposed that IscR might directly sense oxidative stress through the destabilization of its Fe-S cluster [6]. Hydrogen peroxide (H_2_O_2_) was shown to inactivate the *E. coli* Isc system and to activate the *suf* operon through OxyR [18].

Phototrophic bacteria have a special need for the regulation of the Fe-S cluster assembly. Fe-S clusters are required for some components involved in photosynthesis like enzymes for bacteriochlorophyll synthesis (magnesium chelatase and the dark-operative protochlorophyllide oxidoreductase) or the cytochrome bc_1_ complex for photosynthetic electron transport. On the other hand, photooxidative stress caused by the formation of ROS by the light excitation of bacteriochlorophyll destroys Fe-S clusters, which in turn leads to a further increase of ROS levels by the Fenton reaction. Latifi and co-workers suggested that elevated levels of ROS upon iron starvation [19], as also determined for *R. sphaeroides* [20], might be a characteristic for photosynthetic bacteria. Therefore, it is important to study the regulation of Fe-S cluster assembly also in phototrophic bacteria that significantly differ in their lifestyle from *E. coli*.

*R. sphaeroides* is a facultative photosynthetic bacterium, which can use a variety of metabolic pathways for ATP production. At high oxygen tension, aerobic respiration generates ATP. When the oxygen tension in the environment drops, the synthesis of photosynthetic complexes is induced, while aerobic respiration still takes place. Under anaerobic conditions in the light, anoxygenic photosynthesis generates ATP, while under dark conditions in the presence of a suitable electron acceptor, anaerobic respiration is performed. Oxygen is a major regulatory factor for the formation of photosynthetic complexes, and several proteins involved in oxygen-mediated gene regulation have been identified [21,22]. Under iron limitation, *R. sphaeroides* loses its purple color due to the loss of photosynthetic complexes and is no longer able to grow phototrophically [23]. Since bacteriochlorophyll synthesis requires iron, and also the reaction center contains iron, no photosynthetic complexes can be formed under iron limitation.

*R. sphaeroides* serves as a model organism to elucidate the response of photosynthetic bacteria to singlet oxygen [24,25,26,27,28,29] and has also been analyzed in regard to its response to iron limitation in oxic and anoxic environments [20,23,30,31]. The Fur ortholog (Fur/Mur) of *R. sphaeroides* is involved in both iron and manganese homeostasis [23]. Like in other alpha-proteobacteria, the Irr (**i**ron **r**esponse **r**egulator) protein was shown to affect genes of the iron metabolism [30]. Lack of Fur/Mur, as well as lack of Irr, results in stronger induction of *isc/suf* genes under iron limitation [23,30]. Two-thirds of the iron-dependent genes in *R. sphaeroides* showed different responses under oxic or anoxic conditions. For some of these genes, including *isc-suf* genes, induction under iron limitation under oxic conditions was mediated by the OxyR protein [20]. *R. sphaeroides* harbors a large operon comprising *isc* and *suf* genes for iron-sulfur cluster assembly. Like in *E. coli*, the product of the first gene, IscR, functions as an iron-dependent repressor of the *isc* genes [31]. To better understand the transcriptional regulation that adjusts *isc-suf* operon expression to changing environmental conditions, we identified promoters responsible for *isc-suf* expression and quantified their activities under different oxygen concentrations in response to iron limitation and oxidative stress. This study resulted in a complex model for *isc-suf* gene regulation in a phototrophic alpha-proteobacterium.

## 2. Materials and Methods

### 2.1. Bacterial Strains and Growth Conditions

Bacterial strains are listed in Table S1. All *E. coli* strains were cultivated in Standard I medium at 37 °C, either in liquid culture by shaking at 180 rpm or on a solid growth medium, which contained 1.6% (*w/v*) agar. Depending on the cultivated strain, the antibiotics kanamycin (25 μg mL^−1^), ampicillin (200 µg mL^−1^), or tetracycline (20 μg mL^−1^) were added to the liquid and solid growth media.

*R. sphaeroides* strains were cultivated in 50 mL Erlenmeyer flasks containing 40 mL malate minimal medium [20] with continuous shaking at 32 °C (microaerobic growth with a dissolved oxygen concentration of 25–30 μM). Aerobic conditions with 160 to 180 μM dissolved oxygen were achieved by incubating 25 mL of culture in 100 mL Erlenmeyer baffled flasks. The iron-depleted medium used was malate minimal medium without the addition of Fe(III) citrate [20,23] and containing the iron chelator 2,2’-dipyridyl (30 mM, Merck, Darmstadt, Germany). The cells were grown overnight and then transferred to new iron-depleted malate minimal medium three times more before harvesting [20,23]. Cells were harvested at an OD_660_ of 0.4–0.6. Antibiotics were added to the liquid and solid growth media depending on the cultivated strain at the following concentrations: spectinomycin (10 μg mL^−1^), kanamycin (25 μg mL^−1^), gentamicin (25 μg mL^−1^), tetracycline (2 μg mL^−1^). For generating oxidative stress, the cultures of *R. sphaeroides* wild type or mutants were grown in iron-repleted malate minimal medium and treated with 1 mM (final concentration) hydrogen peroxide or 100 μM (final concentration) tertiary butyl-alcohol (tBOOH) for 7 min or 30 min at an OD_660_ of 0.5. After 0, 7, and 30 min, cells were harvested and used for ß-galactosidase measurements.

### 2.2. Construction of R. sphaeroides RirA Deletion Mutants

*R. sphaeroides* strain 2.4.1*Δ*RSP_3341 was generated by transferring the suicide plasmid pPHU2.4.1*Δ*RSP_3341::Sp (RirA homolog 1) into *R. sphaeroides* 2.4.1, and screening for the insertion of the spectinomycin resistance cassette into the chromosome by homologous recombination. Parts of the *rirA* gene (RSP_3341) of *R. sphaeroides*, together with upstream and downstream sequences, were amplified by using oligonucleotides 3341up_f/3341up_r and 3341dn_f/3341dn_r. The amplified PCR fragments were cloned into the *Kpn*I-*BamH*I and *BamH*I-*Hind*III sites of the suicide plasmid pPHU281 [32], generating the plasmid pPHU2.4.1*Δ*RSP_3341. A 2.2 kb *BamH*I fragment containing the spectinomycin cassette from pHP45Ω [33] was inserted into the *BamH*I site of pPHU2.4.1*Δ*RSP_3341 to generate pPHU2.4.1*Δ*RSP_3341::Sp. This plasmid was transferred into *E. coli* strain S17-1 and diparentally conjugated into *R. sphaeroides* 2.4.1 wild-type strain. Conjugants were selected on malate minimal salt agar plates containing spectinomycin. By insertion of the spectinomycin cassette, 438 bp of *R. sphaeroides rirA* gene RSP_3341 was deleted. *R. sphaeroides* strain 2.4.1*Δ*RSP_2888 was generated by transferring the suicide plasmid pPHU2.4.1*Δ*RSP_2888::Km (RirA homolog 2) into *R. sphaeroides* 2.4.1, and screening for insertion of the kanamycin resistance cassette into the chromosome by homologous recombination. Parts of the *rirA* gene (RSP_2888) of *R. sphaeroides*, together with upstream and downstream sequences, were amplified by using oligonucleotides 2888up_f/2888up_r and 2888dn_f/2888dn_r. The amplified PCR fragments were cloned into the *Kpn*I-*BamH*I and *BamH*I-*Hind*III sites of the suicide plasmid pPHU281 [32], generating the plasmid pPHU2.4.1*Δ*RSP_2888. A 2.2 kb *BamH*I fragment containing the kanamycin cassette from pHP45Ω [33] was inserted into the *BamH*I site of pPHU2.4.1*Δ*RSP_2888 to generate pPHU2.4.1*Δ*RSP_2888::Sp. This plasmid was transferred into *E. coli* strain S17-1 and diparentally conjugated into *R. sphaeroides* 2.4.1 wild-type strain. The conjugants were selected on malate minimal salt agar plates containing kanamycin.

### 2.3. Constructions of Promoter Fusion Plasmids

According to the dRNA-seq data, fragments with lengths ranging from 98 bp to 1777 bp containing one of the putative five different single promoters or combined promoters of the *isc-suf* operon, respectively, were amplified by PCR with primers listed in Table S2 The PCR product was ligated into the pJET1.2/blunt cloning vector (Thermo Fisher, Dreieich, Germany) and then transferred into *E. coli* JM109. After confirming the correct sequence, the promoter fragment was cut from the sequenced cloning vector by *Xba*I/*Pst*I or *Spe*I/*Crf*9 and subsequently ligated into the transcriptional *lacZ* fusion vector pBBR1-MCS3-LacZ [34]. For testing the effect of antisense RNA on the activity of P2, the identical promoter fragment was also ligated into the *Xba*I/*Pst*I sites of the transcriptional *lacZ* fusion vector pBBR1-MCS5-LacZ, which harbors a gentamicin cassette and can be transferred into *R. sphaeroides* together with plasmid pRK4352 [35] and its derivatives.

### 2.4. Construction of Altered Promoter Sequences by Site-Directed Mutagenesis

Mutations in promoters of the *isc-suf* operon were constructed by inverse PCR. All primers used are listed in Table S2. For constructions of site-directed mutant P4 and P254, the TTG in the –35 region of the P4 promoter was replaced by AAA by using the plasmids pJET1.2-P4 or pJET1.2-P254 as templates, respectively. Similarly, for the constructions of mutant P5 and P25, the TTG in the –35 region of the P5 promoter was replaced by AAA by using the plasmids pJET1.2-P5 or pJET1.2-P25 as templates, respectively. The PCR products were digested by *Dpn*I and then transferred into JM109. Subsequently, the mutated promoter fragments were cut from the cloning vectors with *Xba*I/*Pst*I and ligated into pBBR-MCS3-LacZ [34].

Overexpression of RNA antisense to promoter P2 and sense promoter 2 fusion plasmids.

To test whether the antisense mRNA affects the activity of sense promoter, a DNA fragment antisense to P2 was amplified by PCR with primers asP2_fwd/rev listed in Table S2. The PCR product was ligated into the pJET1.2/blunt cloning vector and then transferred into *E. coli* JM109. After sequencing, the cloned fragment was cut out by *Xba*I-*BamH*I and subsequently ligated into vector pRK4352, which contains the strong 16S promoter [35].

### 2.5. ß-Galactosidase-Measurements

The ß-galactosidase activity of transcriptional fusions was measured by the hydrolysis of O-nitrophenyl- ß-D-galactopyranoside (ONPG) (Serva, Heidelberg, Germany) and expressed as Miller Units, as described in [36].

### 2.6. Differential RNA-seq

RNA isolation, TEX (Terminator EXonuclease) treatment, library construction and sequencing, read mapping, and transcription start site prediction have been described in detail in [37]. The RNA-seq data sets are available at the NCBI Gene Expression Omnibus database under accession number GSE71844.

### 2.7. RNA Isolation and Quantitative RT-PCR

Twenty milliliters of *R. sphaeroides* cells were harvested by centrifugation when an OD_660_ of 0.5 was reached. Total RNA for quantitative RT-PCR was isolated by using the peqGOLDTriFast kit (Peqlab, Erlangen, Germany), as described by the manufacturer. Remaining traces of DNA were removed by the TURBO DNaseI (Invitrogen, Schwerte, Germany). To further confirm the absence of DNA, PCR was performed targeting *gloB* (RSP_0799) with the primers listed in Table S2. Quantitative RT-PCR was performed in a Bio-Rad CFX96 Real-Time system, as described in our previous study [20]. The reference gene *rpoZ* encoding the ω-subunit of RNA polymerase of *R. sphaeroides* was used to normalize the mRNA expression levels [38] according to the formula given by Pfaffl [39]. Primers are listed in Table S2.

### 2.8. Analysis of isc-suf Operon Synteny

The synteny analysis was performed by applying the EDGAR software [40].

Purification of the IscR, Irr, and OxyR proteins and electrophoretic mobility shift assays with dsDNA and protein.

*E. coli* M15 (pREP4/pQE2.4.1oxyR) was used for the overexpression of His-tagged OxyR protein, and the purification of OxyR was performed, as described earlier [41]. Isolation of His-tagged Irr from *E. coli* M15 (pREP4/pQE2.4.1*irr*) is described in [30], and the purification of His-tagged IscR from *E. coli* M15 (pREP4/pQE2.4.1*iscR*) in [31]. Binding of the proteins to the promoter regions of the *isc-suf* operon was determined by an electrophoretic mobility shift assay, as described previously [30,38]. DNA fragments containing the promoter regions of the *isc-suf* operon were produced by PCR. Primers used in the PCR are listed in Table S2. The final volume of the binding reactions was 15–20 µL. The reaction mixtures contained varying amounts of protein, ~3–7 fmol γ-^32^PATP labeled DNA fragment (2000 cpm, Hartmann Analytik, Braunschweig, Germany), salmon sperm DNA (1 μg), and binding buffer, as described elsewhere [30,42]. After the binding reactions were incubated for 30 min at room temperature or 4 °C, the samples were loaded onto a 4% (*w/v*) polyacrylamide gel in 0.5×TBE buffer (22 mM Tris-HCl, 22 mM boric acid, 0.5 mM EDTA, pH 8.3), and the electrophoresis was performed at 130–180 V for 2–5 h at room temperature or at 4 °C. Irr and IscR were isolated and tested under non-reducing conditions. OxyR was purified and tested under reducing and non-reducing conditions with the same outcome.

## 3. Results

### 3.1. Prediction of Promoters for the isc-suf Operon of R. sphaeroides Based on dRNA-Seq Analysis

**Figure 1:**
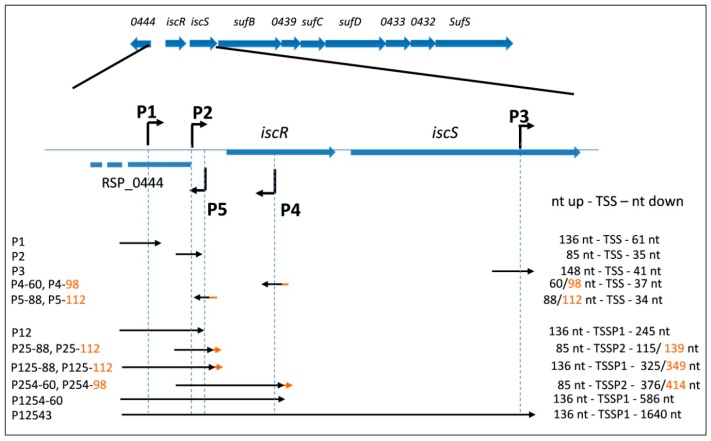
Schematic overview of the *isc-suf* operon of *R. sphaeroides*. The scheme at the top shows the arrangement of *isc-suf* genes and the arrows indicate the five transcriptional start sites (TSS) identified by dRNAseq (Figure S2). Fragments used in the reporter assays to examine the promoter activity for individual promoters and promoter combinations are indicated by the arrows below. The designations for the constructs are given on the left, and the number of nucleotides upstream or downstream of the TSS that are present in the reporter plasmids, are indicated on the right. Upstream or downstream regions of different sizes are marked in orange, and the number of the nucleotides (nt) that extend the shorter DNA fragment is added to the name of the shorter construct. According to the genome annotation of *R. sphaeroides*, the *isc-suf* genes are transcribed from the minus strand. We flipped this orientation in all figures to allow direct recognition of promoter sequences or other motifs.

*R. sphaeroides* harbored a cluster of genes, with *isc* and *suf* homologs (Figure 1). RNA-seq data suggested the co-transcription of these genes, and this was also confirmed by further RT-PCR experiments [31]. The first gene of the *R. sphaeroides isc-suf* operon encoded the IscR regulator, which coordinates a Fe-S cluster with a unique Fe-S ligation scheme [31]. The other genes of the operon encoded two cysteine desulfurases (IscS and SufS), the membrane component of an iron-regulated ABC transporter (SufB), a hypothetical protein (RSP_0439), the ATPase subunit of an ATP transporter (SufC), an Fe-S cluster assembly protein (SufD), and two proteins of the Yip1 family (RSP_0433 and RSP_0432) (Figure 1). This arrangement of genes was highly conserved in the family of *Rhodobacteraceae* (Figure S1). The synteny of genes that were annotated as *isc* or *suf* genes was highly conserved among the anaerobic anoxygenic phototrophs (AnAPs) and aerobic anoxygenic phototrophs (AAPs). There was some variation in regard to the non-annotated open reading frames. The non-phototrophs *Ruegeria* and *Paracoccu*s also showed a similar synteny of *isc* and *suf* genes, and the gene for the IscR homolog of *Paracoccus denitrificans* was, however, positioned at a different chromosomal locus. A similar synteny of *iscS* and *suf* genes was also found in *Rhizobiales* (Figure S1); however, these bacteria lack IscR homologs [43]. The arrangement of genes did not provide information on the organization within an operon. In *Rhodobacter capsulatus*, SB1003 RNAseq data indicated co-transcription of *isc-suf* genes (GEO GSE134200) as in *R. sphaeroides*.

Previous studies addressed the role of proteins with established roles in iron-dependent regulation in *isc-suf* expression in *R. sphaeroides* [23,30,31,36,37]. Different effects of Fur/Mur, Irr, and IscR on individual *iscR* and *suf* genes suggested that not all *isc-suf* genes were under the control of a single promoter and the identical regulatory elements.

Most *R. sphaeroides* promoters share little sequence identity [37], which makes sequence-based promoter identification almost impossible. Therefore, total RNA was isolated from cultures in the exponential growth phase and used for a differential RNA-seq analysis to determine transcriptional start sites (TSS) [37]. This analysis compared RNA, which was treated with terminator exonuclease (TEX) with untreated RNA samples [44]. While TEX degraded RNAs with 5’monophosphate, which were generated by processing, primary transcripts with 5’triphosphate were protected from degradation. Accumulation of the sequencing reads 5’of a gene in the TEX-treated sample, therefore, strongly supported the presence of a TSS at this position. As shown in Figure S2 (overview in Figure 1), the dRNA-seq read coverage indicated the presence of two promoters (P1 and P2) upstream of *iscR*. Another promoter (P3) was predicted within *iscS* and initiated transcripts spanning the *suf* genes. Furthermore, two promoters on the opposite DNA strand might lead to transcripts that were partially antisense to *isc-suf* transcripts. P4 was represented by a very low number of reads and led to a transcript mostly antisense to the *iscR* mRNA. P5 represented the promoter for transcription of RSP_0444. In previous studies, RSP_0444 showed only small differences in expression in the various strains or response to iron-limitation or H_2_O_2_ [23,30,31,36,37]. However, the 5’end of this transcript would partially be antisense to transcripts for the *isc-suf* genes initiating at P1 or P2. The dRNA-seq data did not hint to further promoters for *isc-suf* transcription [31]. Figure 1 shows a schematic overview of the promoter arrangement.

Many promoters in *R. sphaeroides* have a TTG around position –35 and an A at position –10/–11 relative to the TSS [37]. For the putative P1 promoter, an A was in position –11 in regard to the TSS, but no TTG around position –35 was present. The putative P2 promoter had an A residue at position –11 and a TTG around position –35 (Figure S3). Rodionov and co-workers predicted the presence of an Irr box and an IscR box upstream of the *iscR* gene [43]. The predicted IscR box spanned the positions –36 to –19 and the predicted Irr box the positions –13 to +1 in relation to the TSS at P2 (Figure S2, TSS indicated in red). Binding of IscR to the upstream region of the *iscR* gene was experimentally verified previously [31]. For the putative promoter within *iscS* (P3), an A residue at position –10 and a TTG around position –34 were present. Putative promoter P4, which seemed to be very weak based on the read number detected in RNA-seq, had a TTG around position –35, and the same was true for the putative promoter P5.

### 3.2. Activities of the isc-suf Sense and Antisense Promoters Alone or in Combination, as Determined by Transcriptional Reporter Gene Fusions

**Figure 2:**
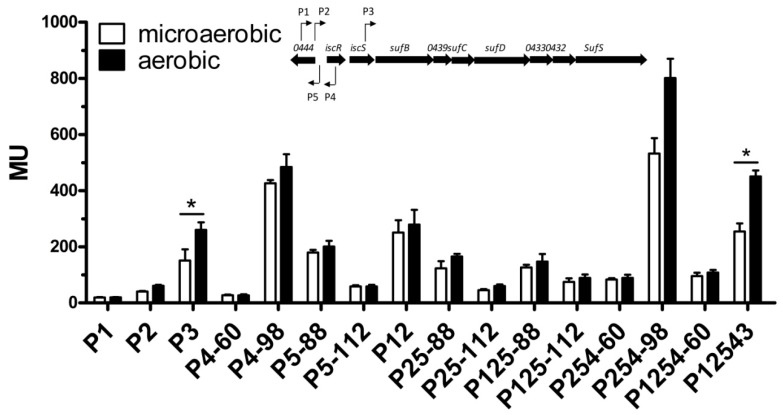
The activity of individual promoters and promoter combinations, as determined by *lacZ* reporter assays and quantified by measuring the ß-galactosidase activity in Miller Units (MU). Cells were cultured under aerobic or microaerobic conditions, as described in Materials and Methods. The scheme at the top shows the arrangement of *isc-suf* genes and the arrows indicate the five transcriptional start sites (TSS) identified by dRNAseq (Figure S2).The designations for all constructs are shown in Figure 1. The bars represent the average of technical duplicates from biological triplicates, and the standard deviation is indicated. *: the difference between the values for iron-replete and iron deplete conditions is >1.5-fold with a *p*-value of <0.01.

In order to confirm the activity of the predicted promoters, we constructed transcriptional fusions to the *lacZ* gene. Fragments containing 60–148 nt upstream and 34–63 nt downstream of the TSS, as determined by dRNA-seq, were cloned in front of the *lacZ* gene of plasmid pBBR1-MCS3-*lacZ* [34] (Table S3). An overview of the cloned fragments is shown in Figure 1, while the exact positions of primers together with predicted TSS are depicted in Figure S3. The plasmids were transferred to *R. sphaeroides* 2.4.1 wild type by conjugation, and the ß-galactosidase activity was determined for exponentially growing cultures. Since the oxygen levels influence the formation of photosynthetic complexes and consequently the demand for Fe-S clusters in *R. sphaeroides*, the cultures were either incubated under aerobic or microaerobic conditions. Under the latter conditions, the formation of photosynthetic complexes was strongly increased. The activity determined for the different promoter fusions showed marked differences (Figure 2). While P1 and P2 exhibited the weak activity of about 20–50 Miller Units (MU), the P3-*lacZ* and P5-88-*lacZ* fusions resulted in about 150–200 MU, the P4-98-*lacZ* fusion in around 400 MU under microaerobic conditions. The high activity of P4 was surprising, considering the fact that only a low number of reads from the P4 promoter was detected by RNA-seq, indicating that the native transcript initiating at P4 might be very unstable in contrast to the P4-*lacZ* fusion. This high activity, however, strongly decreased when the upstream region was reduced from 98 nt to 60 nt (P4–60). Likewise, the activity of P5 was dependent on the length of the upstream region: a shorter upstream region (88 nt) resulted in higher promoter activity than a longer upstream region (112 nt). We describe below that the OxyR protein binds to the P5 upstream region and represses P5 activity. Only for promoter P3, the activity was significantly (increase >1.5-fold and *p* < 0.01) but only slightly higher (slightly more than 1.5-fold) in aerobic conditions compared to microaerobic growth.

Considering the arrangement of the different promoters on the chromosome, it is conceivable that both P1, P2, as well as P3, contribute to *isc-suf* operon transcription and that the antisense transcripts initiating at P4 and P5 may also influence *isc-suf* operon transcription. To test this hypothesis, we applied the same primers as used for the single promoter fusions to also construct reporter plasmids that harbor combinations of the different promoters (Figure 1). Our results confirmed that the presence of additional promoters could influence the expression of the *lacZ* gene, which was fused to one of the promoters (Figure 2). When P1 was present together with P2, the activity was clearly higher than for the single promoter fusions. Remarkably, also the presence of the P5 promoter (RSP_0444 promoter) with 88 nt long upstream sequence, which generated transcripts that were antisense to the 28 nt at the 5’end of the *iscR* transcript, increased P2 activity, but not P12 activity. This activation did not occur, when 112 nt of the P5 upstream region were present (P5–112). The additional presence of the strong P4 promoter led to a strong increase of the activity compared to the P25-*lacZ* fusion, when 98 nt long upstream region was present (P254–98) but not with only 60 nt of the upstream region (P254–60).

When we used the 1777 nt fragment harboring all five promoter regions upstream of *lacZ* (P12543), the ß-galactosidase level was lower than for P254 and similar to P12 under microaerobic conditions (Figure 2). While the P12 activity was independent of the oxygen levels, the P12543 activity was slightly (1.8-fold) but significantly increased under aerobic conditions.

### 3.3. An AntiSense Promoter Stimulates Transcription of the isc-suf Operon

**Figure 3:**
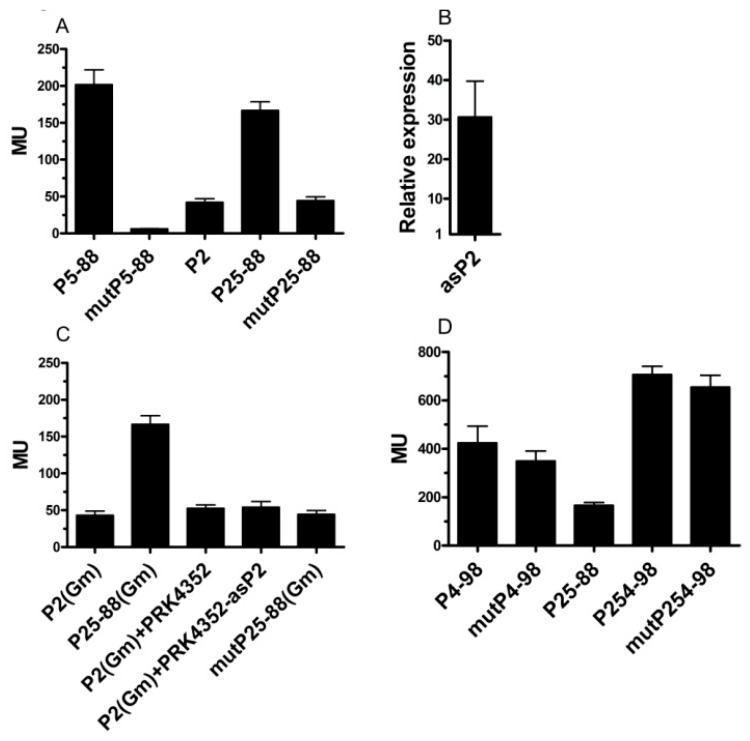
Effects of the antisense promoters P5 and P4 on the activity of P2, as determined by the *lacZ*-reporter assay (ß-galactosidase activity in Miller units). (**A**) The activity of individual promoters P5 or P2 or the combined P25 promoters. The numbers indicate the length of the DNA sequence upstream of the TSS, as shown in Figure 1. “mut” indicates that the TTG at position –35 of promoter P5 was changed to AAA. (**B**) Change of the level of RNA antisense to P2 in a wild type strain harboring the P2 reporter plasmid and plasmid pRK4352-asP2 (overexpression of the antisense RNA) compared to a strain just harboring the P2 promoter. The RNA level was determined by real-time RT-PCR, and the bar represents the average level of technical triplicates from biological triplicates with standard deviation. (**C**) Effect of elevated levels of RNA antisense to P2 on the activity of P5, as determined by the *lacZ*-reporter assay. (Gm) indicates that these reporter constructs carry a gentamycin resistance (all other reporters carry tetracycline resistance) to allow selection of the overexpressed plasmid pRK4352-asP2 (tetracycline resistance). (**D**) The activity of individual promoter P4 or the combined P25 and P254 promoters. The numbers indicate the length of the DNA sequence upstream of the TSS. “mut” indicates that the TTG at position –35 of promoter P4 was changed to AAA.

To study a possible influence of the antisense promoters P4 and P5 (promoter for RSP_0444) on P2 (main promoter for *iscR*) activity, we used different sized downstream fragments of P2 fused to *lacZ*. As shown in Figure 2, our data indicated that both P4 and P5 might influence P2 activity. However, we also had to consider the possibility that the prolonged DNA fragments fused to *lacZ* might contain other elements, which could affect the *lacZ* transcript level. For determining the influence of P5, the P2 downstream DNA fragment fused to *lacZ* was extended by only 80 nt (P25–88) or by 104 nt (P25–112). For testing a putative additional effect by P4, the P25–112 fragment was extended by 237 nt (P254–60) or by 275 nt (P254–98) (Figure 1). To verify that different activities of the P2-reporter versus the P25- and P254- reporters are really due to the promoter activity of P4 and P5, we introduced point mutations into the –35 regions of P4 and P5 promoter regions (see material and methods, TTG changed to AAA) to abolish their activity. Figure 3A shows that the point mutation in the –35 region (mutP5–88) indeed almost abolished the activity of the P5 promoter. While the presence of the wild type P5–88 sequence induced P2 activity, no effect of the mutated P5–88 promoter on the P2 activity was observed, strongly suggesting that the activity of this promoter influenced transcription of the opposite DNA strand (Figure 3A). There were two possibilities, how P5 could influence P2 promoter activity: i) through the production of an antisense transcript, or ii) through changing the local DNA topology by its promoter activity. To discriminate between these possibilities, we introduced a second plasmid (pRK4352-asP2) into the strain harboring the P2-*lacZ* fusion. This plasmid allowed the production of an antisense RNA as produced by P5 from the strong 16S promoter. Real-time RT-PCR proved that in the presence of pRK4352-asP2, the amount of the antisense RNA was about 30-fold higher than in a strain lacking this plasmid (Figure 3B). As shown in Figure 3C, the production of this antisense RNA did not affect the activity of P2. The P2 promoter used in these assays (P2 Gm) was cloned into a different plasmid with gentamicin resistance to allow overexpression of the antisense RNA as P2 from a plasmid with tetracycline resistance.

We applied the same strategy to test the effect of P4 on P2 activity. However, changing the TTG at the putative –35 of P4 only slightly decreased ß-galactosidase activity in the P254–98 construct. Consequently, the resulting construct mutP254–98 would still increase the activity of the P25 fusion (Figure 3D). As already shown in Figure 2, the P4–60 construct had a strongly reduced activity compared to P4–98. The low activity of P4-60 was almost abolished, when the –35 region was mutated. This indicated that the sequences in the –98 to –60 upstream region of the P4 promoter were responsible for the strong activity of P4–98 and the stimulating effect of P4–98 on P25. We were unable to recognize a particular motif that could cause this effect.

### 3.4. Effect of Oxidative Stress and Iron Availability on the Activity of the Promoters of the isc-suf Operon

**Figure 4:**
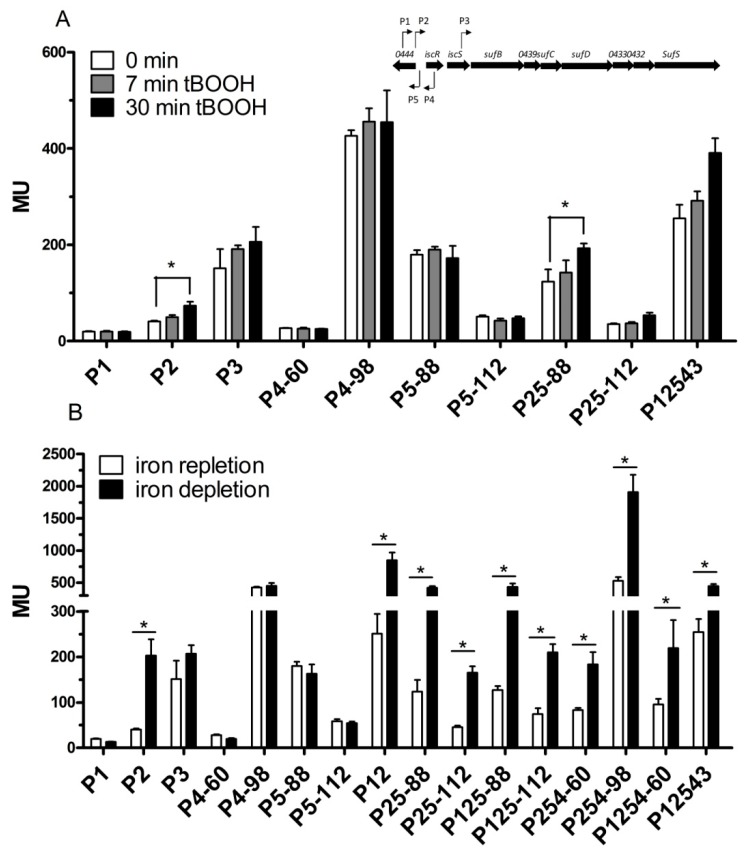
The activity of individual promoters and promoter combinations, as determined by *lacZ* reporter assays and quantified by measuring the ß-galactosidase activity in Miller Units (MU). (**A**) The ß-galactosidase activity was measured before, 7 min, and 30 min after the addition of tBOOH (100 μM final concentration). (**B**) The ß-galactosidase activity was determined under iron repletion and iron depletion. The designations for all constructs are shown in Figure 1. The bars represent the average of technical duplicates from biological triplicates, and the standard deviation is indicated. *: the difference is >1.5-fold with a *p*-value of <0.01.

Previous studies on global gene expression by microarrays or RNA-seq revealed that expression of the *isc-suf* genes is affected by oxidative stress [41,45] and by iron availability [20,23]. In this study, we applied the different reporter fusions to test which promoters are affected by these external factors. We tested hydrogen peroxide and tertiary butyl alcohol (tBOOH) as oxidative stress agents. tBOOH represents organic peroxides, which are generated in the cell, e.g., upon singlet oxygen exposure, and thus represent the photo-oxidative stress that *R. sphaeroides* faces in the presence of pigments, oxygen, and light. None of the promoters P1, P2, P3, P4, P5, or any combination of promoters showed marked changes in activity upon addition of hydrogen peroxide (same values for t0 as shown in Figure 4A and no significant changes at later time points), although hydrogen peroxide was previously shown to strongly induce the *isc-suf* mRNA levels [36]. We had, however, noted before that the response of *lacZ* reporters to H_2_O_2_ might be weak, possibly because of a negative effect on the enzymatic activity [46], while tBOOH was shown to induce the activity of certain *lacZ* fusions [47]. The addition of tBOOH resulted in significantly increased activities of the P2 promoter, and, also, the combination P25–88 showed significant tBOOH-dependent expression (all other promoter fusions did not show altered activity in response to tBOOH). The changes in activity were, however, less than 2-fold (Figure 4A). P12543 was also activated by tBOOH, but the increase was only 1.5-fold.

A previous study demonstrated that the *iscR* transcript level increases upon iron depletion [31]. A strong increase (about 5-fold) of P2 promoter activity upon iron depletion was confirmed in this study and was also observed for all other fusions containing P2, including the long fusion extending to P3 (Figure 4B). No significant effect of iron on activities of P1, P3, P4, or P5 was observed in the wild type (Figure 4B).

### 3.5. Protein Regulators of the isc-suf Operon

Previous bioinformatic analysis predicted IscR and Irr binding sites upstream of the *iscR* gene [43] (Figure S2), and the binding of IscR to this region was experimentally verified [31]. The role of Irr in the expression of the *suf* genes was supported by microarray analysis [30], but binding of the Irr protein to the *iscR* promoter region of *R. sphaer*oides was not reported. Microarray analyses also revealed a stronger effect of iron depletion on *isc-suf* expression in a mutant lacking the Fur/Mur regulator [23] and an effect of OxyR on *isc-suf* expression [18,20]. We tested the activity of the individual *isc-suf* promoters in different mutant strains to get more insights into the influence of these regulatory proteins on the *isc-suf* promoters.

**Figure 5:**
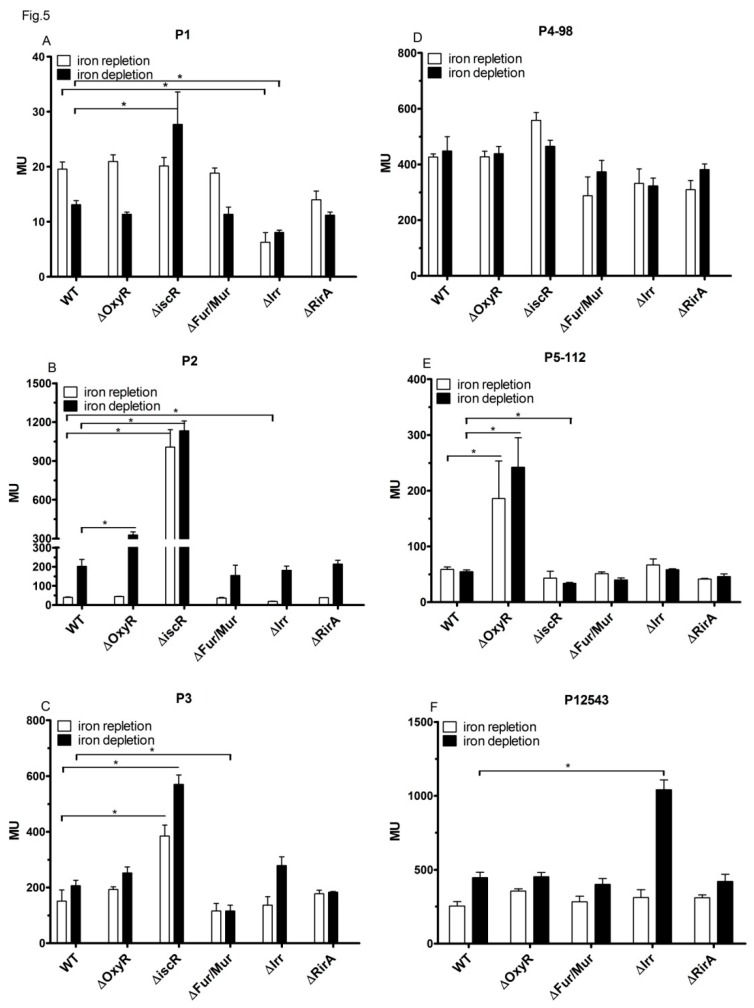
The activity of the individual promoters and P12543, as determined by *lacZ* reporter assays and quantified by measuring the ß-galactosidase activity in Miller Units (MU) in the wild type and mutant strains under iron repletion or iron depletion. (**A**) The activity of promoter P1, (**B**) The activity of promoter P2, (**C**) The activity of promoter P3, (**D**) The activity of promoter P4, (**E**) The activity of promoter P5, (**F**) The activity of P12543. The bars represent the average of technical duplicates from biological triplicates, and the standard deviation is indicated. *: the difference is >1.5-fold with a *p*-value of <0.01.

**Figure 6:**
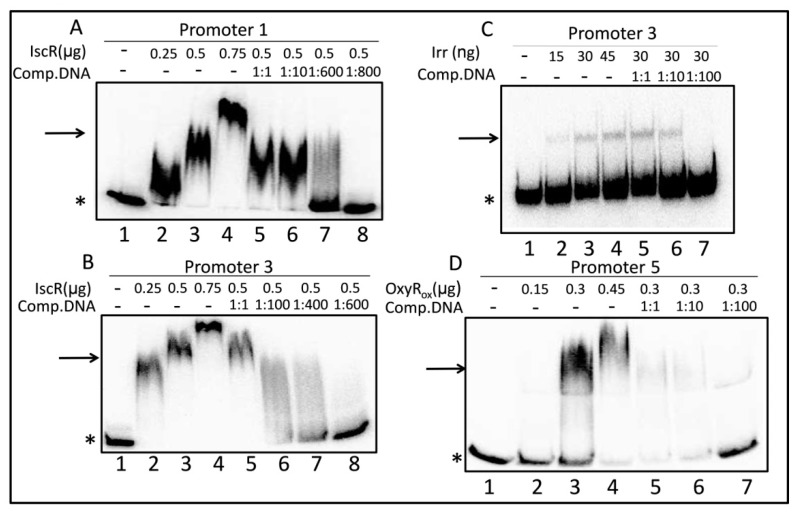
Electrophoretic mobility shift assays showing the interaction of (**A**) IscR to the P1 promoter region (199 bp fragment) and (**B**) IscR to the P3 promoter region (190 bp fragment) and (**C**) Irr to the P3 promoter region (190 bp fragment) and (**D**) oxidized OxyR to the P5 promoter region (147 bp fragment). The star labels the radio-labeled input DNA fragment, and the arrow points to the shifted bands of the DNA protein complexes. The amount of the protein input is given for each lane, as well as the molar ratio of specific, unlabeled competitor DNA (same DNA fragment but without a label).

Only the mutant strains, lacking the IscR or Irr protein, showed the altered activity of the P1 promoter (Figure 5A). The activity of P1 was almost 3-fold lower in the *irr* mutant than in the wild type under iron repletion and 1.6-fold lower under iron depletion. A sequence (TAGAAGGCATAGTGC) with similarity to the consensus Irr-box (Figure S2) was present directly upstream of the TSS of P1. However, we could not confirm binding of Irr to the P1 promoter region in vitro, while in a parallel control assay binding to the *mbf*A promoter, as described in [30], could be observed and confirmed that the isolated Irr protein was able to bind to one of its known targets (Figure S4A). Activity in the *iscR* mutant was similar to that of the wild type under iron repletion but about 2-fold higher under iron depletion. A sequence with some similarity to the *iscR* box (Figure S2) was present at the transcriptional start site (TAGACGACCTTGTTGTT, Figure S3). Indeed, gel retardation revealed that IscR showed specific interaction to the P1 promoter region (Figure 6A). Increasing amounts of IscR protein shifted the P1 containing DNA fragment, while the addition of unlabeled, specific competitor released the shift.

The activity of P2 was strongly influenced by IscR under iron repletion and iron depletion, as demonstrated previously [31] (Figure 5B). The activity of P2 was also elevated in the presence of tBOOH in iron-replete medium (Figure 4A). This response of P2 to tBOOH was lost in the *iscR* mutant (Figure S5). Furthermore, we observed small but significant effects of OxyR and Irr on P2 activity: lack of OxyR increased P2 activity about 1.5-fold under iron depletion, the lack of Irr decreased P2 activity about 2-fold under iron repletion. Binding of IscR to P2, as well as regulation of P2 by IscR, was demonstrated before [31]. An Irr binding site was predicted for the P2 promoter region [43]. As for P1, we could not confirm binding of Irr to the P2 promoter region, while in a parallel control assay, binding to the *mbf*A promoter, as described in [30], could be observed (Figure S4B).

P3 activity was affected by IscR, both under iron repletion and iron depletion (Figure 5C). Lack of IscR resulted in higher activity (2.8-fold) in iron depletion, as well as in iron repletion (2.5-fold), indicating a repressing effect of IscR under both conditions. In the wild type, P3 showed no significant response to iron, while we observed slightly higher activity (1.5-fold) under iron depletion in the strain lacking IscR. Inspection of the sequence around the predicted TSS of P3 revealed a motif (GACTATTTCTGTCGG) with similarity to the consensus IscR box (Figure S2). Indeed, IscR showed specific binding to a DNA fragment containing the predicted binding site in a gel retardation assay (Figure 6B). Furthermore, the activity of the P3 promoter was significantly reduced in the Fur/Mur mutant under iron depletion (Figure 5C). Only in the strain lacking Irr, we had a significantly increased P3 activity (2-fold increase) in iron depletion compared to iron repletion. This agreed with our previous microarray data: the iron dependency of *suf* transcript levels is more pronounced in a strain lacking Irr than in the wild type. A putative Irr binding site (TTAGAAATATTCTAGA) was present about 50 nt upstream of the TSS of P3, which is a similar distance to the TSS as observed for the confirmed Irr target *ccpA* [30]. A gel retardation assay confirmed the specific binding of Irr to this DNA region (Figure 6C).

Differences in activity between wild type and the tested mutants for antisense promoter P4 were small (≤1.5-fold) and/or statistically not significant for the reporter construct with 98 nt upstream of the TSS (Figure 5D) and also for the construct with only 60 nt of the upstream region.

The activity of antisense promoter P5 was strongly affected by the OxyR protein when 112 nt upstream of the TSS was present. The activity in the mutant was increased by a factor of 3-4 under both iron repletion and iron depletion (Figure 5E). OxyR did not affect P5 activity when only 88 nt of the upstream sequence was present. None of the other tested mutants showed significantly altered P5 activity. Despite the strong differences in the activity of P5 in the presence or absence of OxyR, P5 did not show a response to high oxygen levels (Figure 2) or tBOOH (Figure 4A). We tested whether OxyR was able to bind to the P5 promoter by the gel retardation assay. As shown in Figure 6D, the addition of increasing amounts of oxidized OxyR resulted in retardation of a 147 nt DNA fragment harboring the P5 promoter region fragment. The same result was achieved with reduced OxyR protein. The addition of an excess of the same, but un-labeled DNA fragment led to a decrease of retardation due to specific competition with the labeled fragment for binding. This result strongly supported that OxyR was indeed binding to the P5 promoter region.

We also tested the activity of the P12543 fusion in different mutant strains (Figure 5F) to see how the action of the regulators on promoters P1, P2, P4, or P5 would influence P3 expression. Fur/Mur and the RirA proteins did not influence P12543 activity, as this was also the case for P3 alone. Since the P12543 fusion comprises an intact copy of the *iscR* gene and may, therefore, increase IscR levels in the cell, it could not be tested in conditions lacking IscR. The repressing effect of Irr under iron depletion was more pronounced when all promoters were present than in the P3-fusion alone (compare Figure 5C–F).

### 3.6. The RirA Proteins of R. sphaeroides Have No Effect on isc-suf Expression in R. sphaeroides

While Fur is the dominant iron regulator in gamma-proteobacteria, other proteins have important roles in iron-dependent regulation in alpha-proteobacteria [43]. Besides IscR, another transcriptional regulator of the Rrf2 protein family, RirA, was identified to have an important role in iron regulation in *Rhizobia* [48,49]. The genes RSP_2888 and RSP_3341 of *R. sphaeroides* 2.4.1 have 59%–63% identity to RirA from *Rhizobium leguminosarum* or *Agrobacterium tumefaciens* (*Rhizobium radiobacter*). We constructed knock out strains of *R. sphaeroides* lacking either RSP_2888 or RSP_3341 or both genes together. As seen in Figure 5A–F and Figure S6, the promoters of the *isc-suf* operon showed very similar activities in the wild type and the double mutant, and also the effect of iron depletion on promoter activity was very similar in both strains. We concluded that the RirA homologs of *R. sphaeroides* had no major role in the iron-dependent regulation of the *isc-suf* operon. The double mutant showed identical growth curves as the wild type in iron repletion and iron depletion (Figure S7), indicating that the RirA proteins have also no major impact on iron regulation in *R. sphaeroides* in general.

## 4. Discussion

The assembly of iron-sulfur clusters is an important cellular process in almost all living organisms, and stress conditions that destroy iron-sulfur clusters increase the need for iron-sulfur cluster assembly. The facultative phototrophic bacterium *R. sphaeroides* harbors a large operon that comprises *isc* and *suf* genes, which are distributed to different operons in *E. coli*. Unlike *E. coli*, *R. sphaeroides* can form photosynthetic complexes that cause an additional demand for the Fe-S cluster and can also cause photooxidative stress that destroys the Fe-S cluster. The synteny of *isc* and *suf* genes is conserved among *Rhodobacteraceae* independently of the ability to perform photosynthesis and is also similar in *Rhizobiales*. This suggests that the combined *isc-suf* operons arose early in evolution, in a common ancestor of these orders.

**Figure 7:**
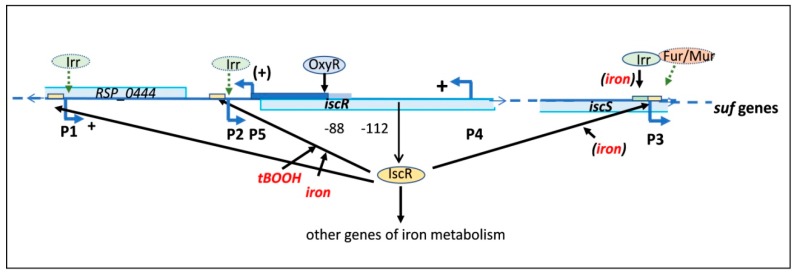
A schematic model, combining the influence of other promoters on P2 and the action of different proteins on the activity of the *isc-suf* promoters. **+** indicates a stimulating effect of P1, P4, and P5 promoter regions (dependent on the length of the upstream region) on P2 activity. The IscR protein binds to IscR boxes (yellow bars) in the promoter regions of P1, P2, and P3, and OxyR binds upstream of the P5 promoter (indicated by solid arrows), and Irr binds to an Irr-box (green bar) in the P3 promoter region. At P2, IscR mediates the response to iron and tBOOH. The iron-dependent activity of P3 is only observed in the absence of IscR or the absence of Irr. Green arrows indicate activation by the protein regulators, and black arrows indicate repression. The small effects of Irr on P1 and P2 and of Fur/Mur on P3 are most likely indirect and do not involve direct binding (indicated by dashed arrows).

Previous work revealed iron- and hydrogen peroxide-dependent expression of the *isc-suf* genes of *R. sphaeroides* and the role of OxyR, Fur/Mur, Irr, and IscR regulators in *isc-suf* expression [20,23,30,31]. To verify promoter activities as predicted by dRNA-seq (Figure S2) and to understand the contribution of the individual promoters, we analyzed activities of individual promoters and of promoter combinations and the effect of iron, oxygen, and oxidative stress on the activities by using reporter gene fusions. These data were combined in a model (Figure 7) that visualizes the complex regulatory network controlling *isc-suf* expression in *R. sphaeroides*.

Our data supported the view that both P1 and P2 contributed to *isc-suf* expression, and P3 further contributed to transcription of the *suf* genes. P3 was the strongest of the sense promoters, and extending the upstream region of P3 and including P1 and P2 elevated the activity further and conferred iron-dependent expression. An additional promoter for the *suf* genes (P3) might guarantee the high expression of the proteins required for the assembly of iron-sulfur clusters. IscR has a regulatory function and may not be required in a high amount. IscS is a cysteine desulfurase that is required for mobilization of sulfur from L-cysteine. A higher expression of *iscS* might not be required, since, with SufS, another cysteine desulfurase was encoded by the *isc-suf* operon downstream of P3. This arrangement with two cysteine desulfurases was conserved among *Rhodobacteraceae* and also found in *Rhizobiaceae* (Figure S1).

Two antisense promoters were present downstream of P2. P4 was located within the *iscR* gene, while P5 was located upstream of *iscR* and responsible for transcription of RSP_0444. The gene product of RSP_0444 is annotated as a putative hydrolase, but no experimental data on its function is available. The position of this gene on the chromosome was also found in most *Rhodobacteraceae* and *Rhizobiaceae*, although in *Rhizobiaceae, iscR* was not located upstream of IscS (Figure S1). Surprisingly, both antisense promoter regions, P5 and P4, positively affected transcription in sense direction. Transcription of *iscR* from P2 was higher, when, at the same time, P5 was present, and even higher, when, also, the region containing P4 was present. RNA-seq analyses revealed the presence of remarkably high numbers of antisense promoters in many bacterial species, and different effects of antisense transcripts have been reported [50,51,52]. In many cases, antisense transcription produces non-coding sRNAs that either interfere with translation or influence stability of the sense RNA. We could not confirm the existence of a distinct, small RNA originating at P4 by Northern blot analysis. Alternatively, the formation of an open complex during initiation of transcription might also allow more efficient transcription in the opposite direction. An effect of promoters on superhelicity-dependent processes is well documented [53]. The exact mechanisms by which P5 and the P4 region affect *isc-suf* expression remain elusive, but our data emphasized that it is important to consider the effect of such antisense promoters for sense promoter activity.

P2 was the main promoter for transcription of *iscRS*, and binding of IscR to P2 conferred iron-dependent expression to P2 and to the downstream promoter P3 that initiated further *suf* transcripts. Our present study revealed that IscR was also required for the tBOOH-dependent activity of P2. Thus, IscR could function as a sensor for iron availability and organic peroxide stress in *R. sphaeroides*. Furthermore, we could demonstrate the binding of IscR to the other sense promoters—P1 and P3. In contrast to its effect on P2 activity, IscR binding to P1 or P3 did not mediate tBOOH-dependent expression. Two types of IscR binding sites—Type 1 and Type 2—were identified in *E. coli*: holo-IscR (containing the Fe-S cluster) shows a higher affinity to Type 1 sites than apo-IscR, while both IscR forms bind with similar affinity to Type 2 sites [9]. Interestingly, this regulatory mechanism is even conserved in the Gram-positive *Thermincola potens* [54]. Our in vivo data revealed stronger repression of the P2 promoter by holo-IscR, while the repressing effect on P3 was similar in iron repletion and iron depletion, and only apo-IscR had a repressing effect on P1. This indicated the presence of different types of IscR binding sites also in *R. sphaeroides*.

Despite the presence of an Irr box around the P2 promoter, we detected only a small effect of Irr on P2 (1.8-fold) and did not detect binding of Irr to the P2 region in vitro. A small effect of Irr on P1 activity (1.5-fold) was observed, but no binding of Irr to the promoter region in vitro was observed. Our results implied that Irr did not make a major contribution to P1 and P2 regulation under the tested conditions and suggested that the influence of Irr might be indirect. There was also an influence of Irr on P3 that was even more pronounced when all upstream promoters were present, and binding of Irr to P3 was demonstrated. Since the P12543 reporter also carries a complete copy of *isc*R, we could not exclude that elevated levels of IscR influence activity of the P3 promoter in this strain. Higher IscR levels could increase the effect of iron in the *irr* deletion strain (compare 5C to 5F). The P3 promoter was the only promoter that showed a significant but small effect in the mutant lacking Fur/Mur, indicating an activating function of Fur/Mur under iron depletion. Johnston and co-workers suggested that iron-dependent regulation in alpha-proteobacteria mainly occurs by regulators different from Fur [55]. Some Fur homologs in *Rhizobia* and the Fur homolog of *R. sphaeroides* were shown to affect the expression of the *sit* operon in an Mn^2+^-dependent manner and were consequently named Mur [23,56,57,58]. The deletion of the *fur/mur* gene resulted in stronger expression of many iron-dependent genes in *R. sphaeroides* [23], suggesting that Fur/Mur has a role in regulating iron metabolism in this bacterium and has a repressing function under iron depletion. Rodionov and co-workers suggested a Fur-box and a slightly differing Mur-box as consensus sequences for DNA binding sites in alpha-proteobacteria [43]. Such sequences are not present in the vicinity of P3 of the *isc-suf* operon, while an almost perfect Mur box is located between the –10 and –35 region of the *sitA* promoter. Fur/Mur is required for a strong induction (about 50-fold) of *sitA* expression in response to Mn^2+^ limitation [23]. It is likely that the small effects of Fur/Mur on many genes of iron metabolism, including P3 of the *isc-suf* operon, does not include direct binding but is rather mediated indirectly.

While none of the sense promoters was influenced by the OxyR protein, antisense promoter P5 was strongly repressed by this regulator, under iron-replete and iron deplete conditions, and binding of OxyR to the P5 upstream region was confirmed. The repressing effect of OxyR on the P5 promoter required 112 nt of the upstream region and was not present with only 88 nt of the upstream region (Figure 5E). An effect of OxyR on the iron-dependent levels of *isc* and *suf* genes was reported previously [20,41,45]. Since the activity of P12543 was not influenced by OxyR (Figure 5F), it was likely that other mechanisms than binding to P5 were responsible for the OxyR effect on the *suf* genes, which might also be indirect. Since P5 is the promoter for RSP_0444, OxyR could also affect the expression of RSP_0444, but previous microarray studies revealed hydrogen peroxide- and OxyR-independent expression of RSP_0444 [41,45]. OxyR is mostly known as an activator of gene expression in response to oxidative stress. A repressor function of OxyR under non-stress conditions, as observed for P5, was, however, also described in *R. sphaeroides* [41].

## 5. Conclusions

Figure 7 summarizes the effects of external factors and regulatory proteins on *isc-suf* expression and also indicates the influence of the P1, P5, and the P4 promoter region on the activity of P2, which is the main promoter for *iscRS* expression but also regulates other genes of iron metabolism. Our study revealed IscR as the main regulator for iron- and tBOOH-dependent expression of the *isc-suf* operon. IscR (under iron repletion and iron depletion) and Irr (under iron depletion) also repressed P3 activity by direct binding. The presence of the upstream region, including P1, allowed higher transcription rates of *isc-suf* than P2 alone, and both antisense promoters stimulated P2 activity. The influence of Fur/Mur was observed for P3, but the effects were small and most likely indirect. Thus, multiple promoters and multiple regulators were involved in adjusting *isc-suf* expression to environmental conditions, and by regulating *iscR* expression indirectly, other genes of the iron metabolism that were controlled by IscR were also affected.

## Supplementary Materials

The following are available online at https://www.mdpi.com/2076-2607/7/12/671/s1.
