# Supplementary material for: Multiple Sense and Antisense Promoters Contribute to the Regulated Expression of the isc-suf Operon for Iron-Sulfur Cluster Assembly in Rhodobacter
Source: Microorganisms. 2019 Dec 10;7(12):671. doi: 10.3390/microorganisms7120671 (PMC6956336; doi:10.3390/microorganisms7120671)
Supplement: Supplementary file 1 [file microorganisms-07-00671-s001.pdf]

Fig. S1

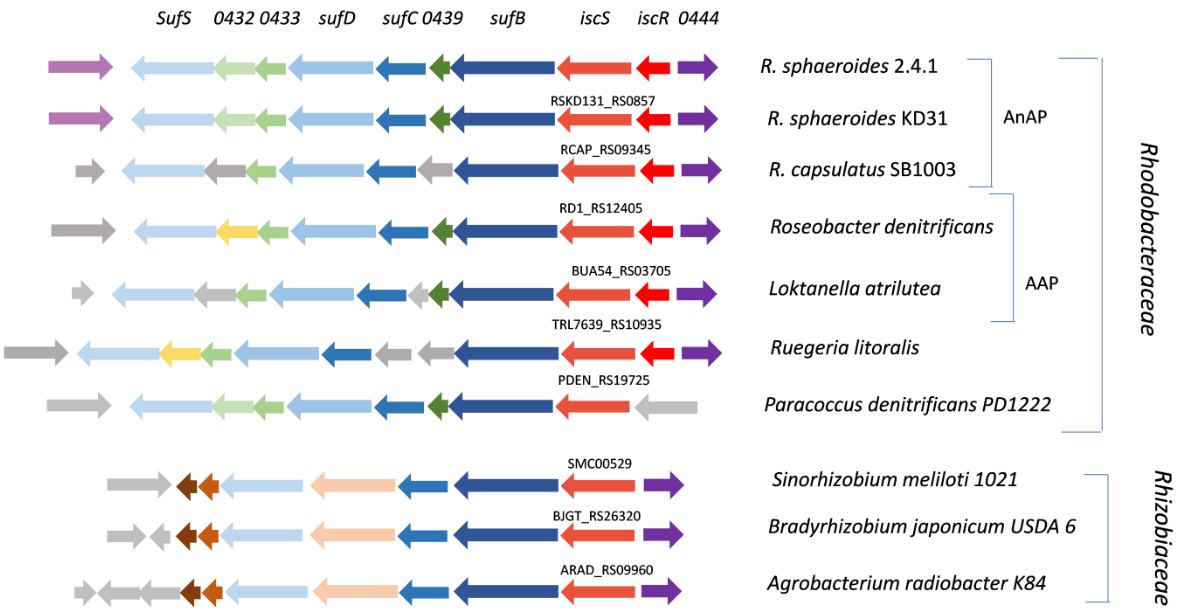

Fig. S1: Overview of the synteny of *suf* genes in selected bacterial genomes. All selected *suf* genes are annotated on the minus DNA strand and the figure displays the genome locations as displayed by genome browsers (in Fig. 1 and Fig. S2 and Fig. S3 the orientation was flipped to recognize promoter sequences and conserved protein binding motifs). Identical colors are used for the orthologs in different species. Grey color indicates no significant homology to the genes in the other species shown in the figure. AnAP: anaerobic anoxygenic phototrophs, AAP: aerobic anoxygenic phototrophs.

Fig. S2

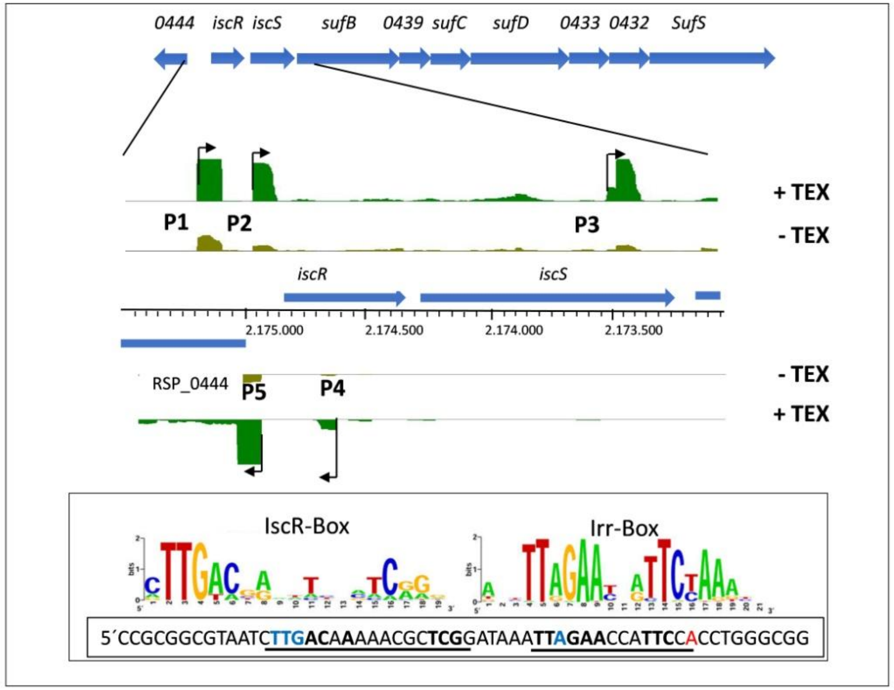

Fig. S2: Schematic overview of the *isc-suf* operon of *R. sphaeroides* and reads from RNA-seq for its 5´region as displayed in the Integrated Genome Browser. + TEX: RNA samples were treated with terminator exonuclease, -TEX samples were not treated. At the bottom the consensus IscR and Irr boxes as presented by [1] are shown together with the sequence of the P2 promoter (shown in plus orientation). The IscR and Irr boxes are underlined, matching bases are shown in bold. The transcriptional start site is shown in red, the A at position -11 and the TTG around -35 are shown in blue. According to the genome annotation of *R. sphaeroides* the *isc-suf* genes are transcribed from the minus strand. We flipped this orientation in all figures to allow direct recogition of promoter sequences or other motifs.

Fig. S3

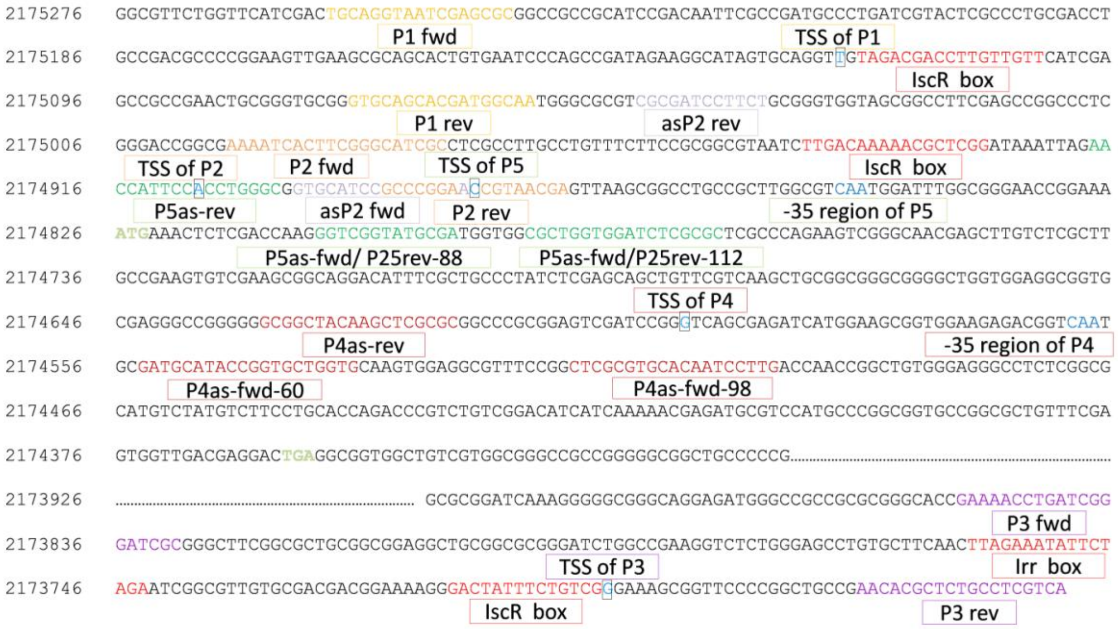

Fig. S3: Sequence of the 5´region of the *isc-suf* operon from *R. sphaeroides* shown in plus direction. The TSS for promoters P1-P5 are indicated (lightblue, boxed), as well as the primer binding sequences for amplification of DNA fragments for reporter plasmid construction. The ATG and the TGA of the IscR coding sequence are marked in bold green. The TTGs at position -35 of P4 and P5 that were mutated to AAA for our investigations are marked in darkblue. asP2rev and asP2fwd mark position of primers for generating RNA anti-sense to P2 as shown in Fig. 3B. We flipped the orientation in this figure to allow direct recogition of promoter sequences or other motifs.

Fig. S4

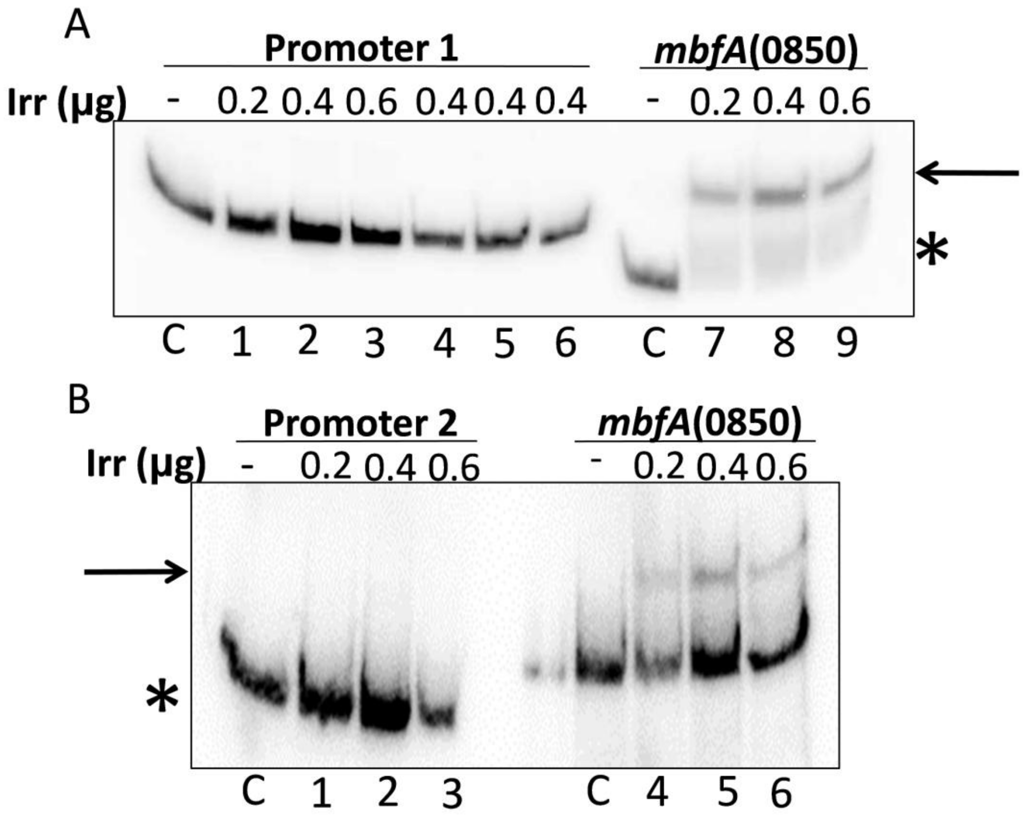

Fig. S4: Electrophoretic mobility shift assays testing the interaction of A: Irr to the P1 promoter region (199 bp fragment) and B: of Irr to the P2 promoter region (147 bp fragment). The promoter region of the *mbfA* gene (180 bp fragment) that is known to bind Irr [2] was used as a positive control. The star labels the radiolabeled input DNA fragment, the arrow points to the shifted bands of the DNA protein complexes. The amount of the protein input is given for each lane and the molar ration of specific, unlabelled competitor DNA.

Fig.S5

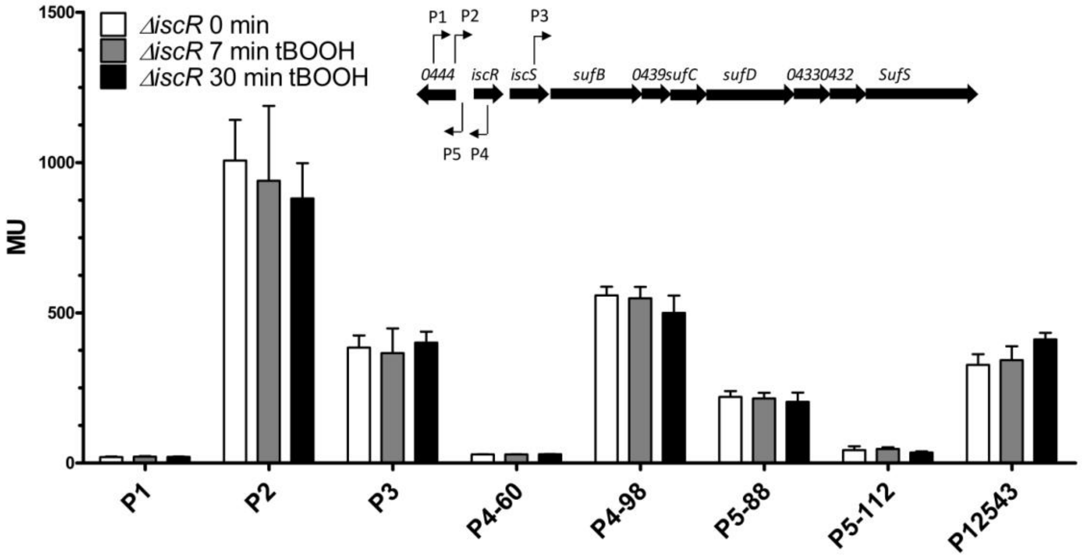

Fig. S5: Activity of individual promoters and promoter combinations as determined by *lacZ* reporter assays and quantified by measuring the β-galactosidase activity in Miller Units (MU). β-galactosidase activity was measured before, 7 min, and 30 min after addition of tBOOH (100 μM final concentration) to the IscR mutant. The bars represent the average of technical duplicates from biological tripliates and the standard deviation is indicated.

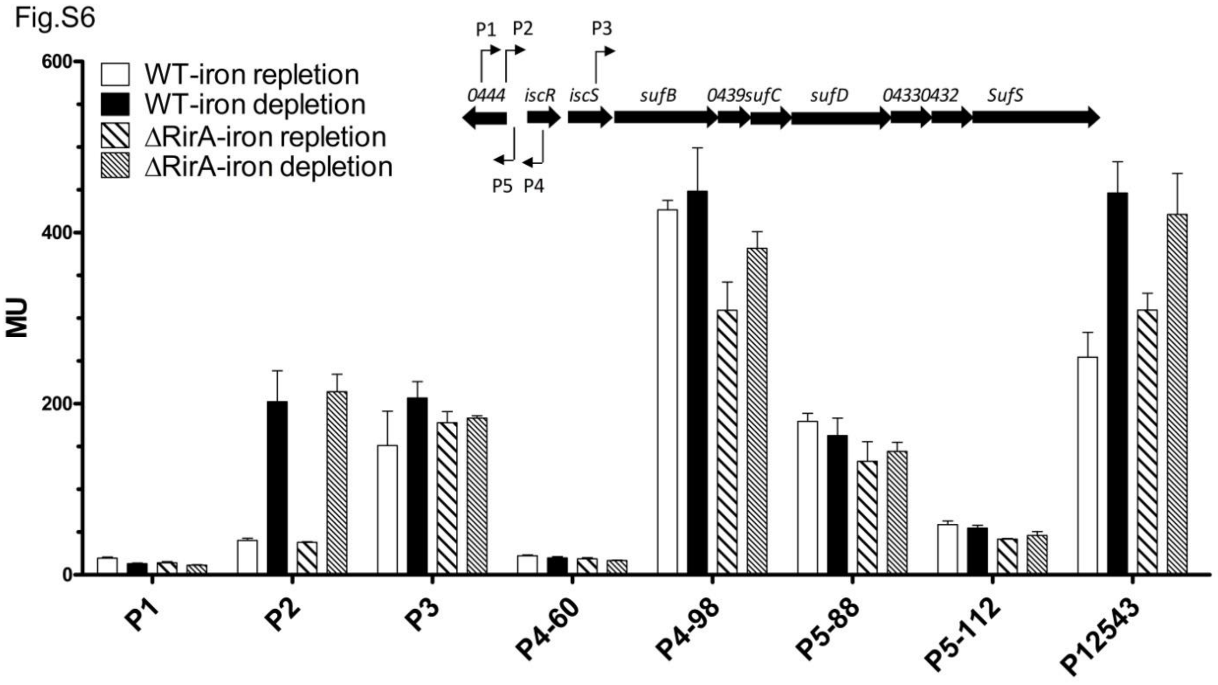

Fig. S6: Activity of individual promoters and promoter combinations as determined by *lacZ* reporter assays and quantified by measuring the β-galactosidase activity in Miller Units (MU). β-galactosidase activity was compared for the wild type and the RirA double mutant under iron repletion and iron depletion. The bars represent the average of technical duplicates from biological tripliates and the standard deviation is indicated.

Fig. S7

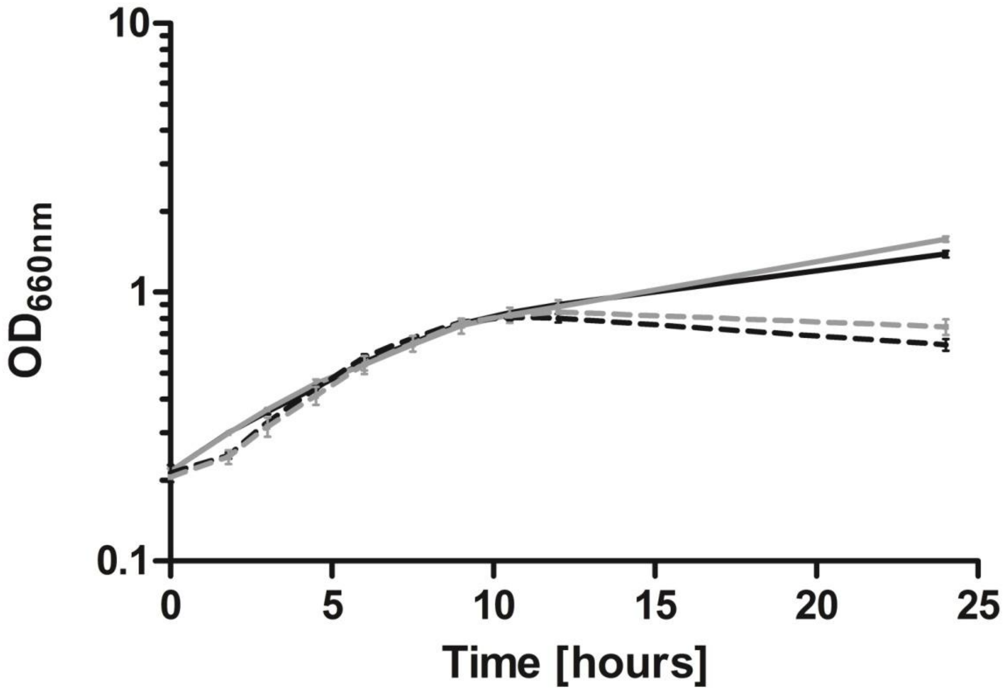

Fig. S7: Growth curves for the wild type and the RirA double mutant under iron repletion and iron depletion. Growth curves of the *R. sphaeroides* wild type (black) and the RirA double mutant (gray) under iron repletion (continuous line) and under iron depletion (dashed line) conditions are shown. The optical density at 660 nm (OD_{660nm}) of microaerobically grown *R. sphaeroides* cultures was determined over time. The data represent the mean of three independent experiments and error bars indicate standard deviation of the mean.

Table S1. *R. sphaeroides* and *E. coli* strains used in this study

**Strains**	**Relevant features**	**References**
R.sphaeroides		
2.4.1	*Rhodobacter sphaeroides* wild type	[3]
2.4.1 Δ*iscR*	Sp^r, *iscR* deletion strain	[4]
2.4.1 Δ*fur/mur*	Sp^r, *fur/mur* deletion strain	[5]
2.4.1 Δ*oxyR*	Sp^r, *oxyR* deletion strain	[6]
2.4.1 Δ*irr*	Km^r, *irr* deletion strain	[2]
2.4.1 ΔRSP_2888	Km^r, *rirA* homolog deletion strain	This study
2.4.1 ΔRSP_3341	Sp^r, *rirA* homolog deletion strain	This study
2.4.1 ΔRSP_2888+3341 (RirA)	Km^r, Sp^r, *rirA* homolog double deletion strain	This study
E. coli		
JM109	Host strain for cloning procedures	[7]
S17-1	Strain for diparental conjugation, tra^+	[8]
M15(pREP4/pQE2.4.1*oxyR*)	M15 containing pQE30::*oxyR*, Km^r, Ap^r, used for OxyR overexpression	[9]
M15(pREP4/pQE2.4.1*iscR*)	M15 containing pQE30::*iscR*, Km^r, Ap^r, used for IscR overexpression	[4]
M15(pREP4/pQE2.4.1*irr*)	M15 containing pQE30::*irr*, Km^r, Ap^r, used for Irr overexpression	[2]

Sp^r, spectinomycin-resistant; Ap^r, ampicillin-resistant; Km^r, kanamycin resistant; when required, antibiotics were added in the following concentrations: spectinomycin (10 μg·ml^{-1}) and kanamycin (25 μg·ml^{-1}) for *R. sphaeroides*; ampicillin (200 μg·ml^{-1}) and kanamycin (25 μg·ml^{-1}) for *E. coli*

Table S2 – Oligonucleotides used in this study

Name	Sequence	Purpose
P1_fwd	ACTATCTAGATGCAGGTAATCGAGCGC	forward primer for promoter 1 of *isc-suf*-operon cloning
P1_rev	ACTACTGCAGTTGCCATCGTGCTGCAC	reverse primer for promoter 1 of *isc-suf*-operon cloning
P2_fwd	ACTATCTAGAAAATCACTTCGGGCATCGC	forward primer for promoter 2 of *isc-suf*-operon cloning
P2_rev	ACTACTGCAGTCGTTACGGTTCCGGGC	reverse primer for promoter 2 of *isc-suf*-operon cloning
P25_rev	ACTACTGCAGGCGCGAGATCCACCAGC	reverse primer for promoter 25(88 nt upstream) of *isc-suf*-operon cloning
P3_fwd	ACTACCCGGGAAAACCTGATCGGGATCGC	forward primer for promoter 3 of *isc-suf*-operon cloning
P3_rev	ACTACTGCAGTGACGAGGCAGAGCGTGTT	reverse primer for promoter 3 of *isc-suf*-operon cloning
P4as_fwd	ACTATCTAGACAAGGATTGTGCACGCGAG	forward primer for promoter 4(98 nt upstream) of *isc-suf*-operon cloning
P4as_rev	ACTACTGCAGCGGCTACAAGCTCGCGC	reverse primer for promoter 4 of *isc-suf*-operon cloning
newP4as_fwd	ACTATCTAGACACCAGCACCGGTATGCATC	new forward primer for promoter 4(60 nt upstream) of *isc-suf*-operon cloning
newP4as_fwd(PstI)	ACTACTGCAGCACCAGCACCGGTATGCATC	new forward primer for promoter 4(60 nt upstream) of *isc-suf*-operon cloning (with PstI)
P5as_fwd	ACTATCTAGAGCGCGAGATCCACCAGC	forward primer for promoter 5(112 nt upstream) of *isc-suf*-operon cloning
P5as_rev	ACTACTGCAGAACCATTCCACCTGGGCG	reverse primer for promoter 5 of *isc-suf*-operon cloning
newP5as_fwd	ACTATCTAGATCGCATACCGACCCTTGG	new forward primer for promoter 5(88 nt upstream) of *isc-suf*-operon cloning
new-P12345_fwd	ACTAACTAGTTGCAGGTAATCGAGCGC	forward primer for promoter 12543 of *isc-suf*-operon cloning
new-P12345_rev	ACTACCCGGGTGACGAGGCAGAGCGTGTT	reverse primer for promoter 12543 of *isc-suf*-operon cloning
asP2_fwd	ACTATCTAGATTCCGGGCGGATGCAC	forward primer for antisense of promoter 2 of *isc-suf*-operon cloning
asP2-rev	ACTAGGATCCCGCGTCGCGATCCTTCT	reverse primer for antisense of promoter 2 of *isc-suf*-operon cloning
RT-asP2_fwd	TTCCGGGCGGATGCAC	forward primer for antisense of promoter 2 of *isc-suf*-operon RT-PCR
RT-asP2-rev	CGCGTCGCGATCCTTCT	reverse primer for antisense of promoter 2 of *isc-suf*-operon RT-PCR
RT_RSP_1669_A	ATCGCGGAAGAGACCCAGAG	forward primer for RSP_1669 (*rpoZ*) real-time RT-PCR
RT_RSP_1669_B	GAGCAGCGCCATCTGATCCT	reverse primer for RSP_1669 (*rpoZ*) real-time RT-PCR
RT_RSP_0799_A	GAA CAA TTA CGC CTTCTC	forward primer for RSP_0799 (*gloB*) real-time RT-PCR
RT_RSP_0799_B	CAT CAG CTG GTA GCT CTC	reverse primer for RSP_0799 (*gloB*) real-time RT-PCR

3341up_r	ACTAGGATCCGTAGATCGAGGCGGTCTC	reverse primer for knock-out of RSP_3341-RirA Homolog 1
3341dn_f	ACTAGGATCCTTCATGGACACGCTCG	forward primer for knock-out of RSP_3341-RirA Homolog 1
3341dn_r	ACTAAAGCTTACATCAACCCGCTGTTCAGC	reverse primer for knock-out of RSP_3341-RirA Homolog 1
2888up_f	ACTAGGTACCGTAGCAAAAGCTGTCCGAG	forward primer for knock-out of RSP_2888-RirA Homolog 2
2888up_r	ACTAGGATCCTCATCGCGAGATTGGTG	reverse primer for knock-out of RSP_2888-RirA Homolog 2
2888dn_f	ACTAGGATCCTTCTACGGCACGCTCGA	forward primer for knock-out of RSP_2888-RirA Homolog 2
2888dn_r	ACTAAAGCTTACGAGGAGATCGGCCTCG	reverse primer for knock-out of RSP_2888-RirA Homolog 2
3341_670upst_f	ACGACGAAACTCGCGGAAGACG	control forward primer for knock-out of RSP_3341-RirA Homolog 1
3341_721upst_f	CGAGATCTTCGGGGTGAGC	control forward primer for knock-out of RSP_3341-RirA Homolog 1
3341_dn2_r	GGTCAACTGGGGGATCTATGTCTG	control reverse primer for knock-out of RSP_3341-RirA Homolog 1
2888_675upst_f	GAACCAGGGCTCCATGATCC	control forward primer for knock-out of RSP_2888-RirA Homolog 2
2888_630upst_f	CATCCGCCAGTCATAGAGGCT	control forward primer for knock-out of RSP_2888-RirA Homolog 2
2888_dn2_r	GGCCAAGACGATCCGCTATT	control reverse primer for knock-out of RSP_2888-RirA Homolog 2
Prom5_TTG_to_AAA	AAATCCAAAAACGCCAAGCGGCAGGCCG	forward primer for rolling cycle/inverse PCR for mutation of P5 and P25 of *isc-suf* operon
Prom5_TTG_to_AAA	TGGCGTTTTTGGATTTGGCGGGAACCGG	reverse primer for rolling cycle/inverse PCR for mutation of P5 and P25 of *isc-suf* operon
Prom254_AAC-TTT	AGACGGTTTTTGCGATGCATACCGGTGC	forward primer for rolling cycle/inverse PCR for mutation of P4 and P254 of *isc-suf* operon
Prom254_AAC-TTT	ATCGCAAAAACCGTCTCTTCCACCGCTT	reverse primer for rolling cycle/inverse PCR for mutation of P4 and P254 of *isc-suf* operon

Table S3 - plasmids used in this study

Plasmid names	Relevant features	Source
pJET1.2/ blunt	Ap^r, 2.97 kb, PCR cloning vector	Fermentas
pPHU281	Tc^r, *lacZ mob*(RP4), suicide vector for knock-out construction	[10]
PRK4352	Tc^r, 16S vector with terminator for antisense promoter construction	[11]
PRK4352-asP2	Tc^r, Antisense of Promoter 2 from *isc suf* operon on PRK4352	This study
pBBR1-MCS5-*lacZ*	Gm^r, Broad-host-range cloning vector	[12]
pBBR1-MCS5-*lacZ*-P2	Gm^r, Promoter 2 from *isc suf* operon on pBBR1-MCS5-*lacZ*	This study
pBBR1-MCS5-*lacZ*-P25-88	Gm^r, Promoter 2 and 5 (88 nt upstream) from *isc suf* operon on pBBR1-MCS5-*lacZ*	This study
pBBR1-MCS3-*lacZ*	Tc^r, broad-host-range cloning vector	[12]
pBBR1-MCS3-*lacZ*-P1	Tc^r, Promoter 1 from *isc suf* operon on pBBR1-MCS3-*lacZ*	This study
pBBR1-MCS3-*lacZ*-P2	Tc^r, Promoter 2 from *isc suf* operon on pBBR1-MCS3-*lacZ*	This study
pBBR1-MCS3-*lacZ*-P3	Tc^r, Promoter 3 from *isc suf* operon on pBBR1-MCS3-*lacZ*	This study
pBBR1-MCS3-*lacZ*-P4-60	Tc^r, Promoter 4(60 nt upstream) from *isc suf* operon on pBBR1-MCS3-*lacZ*	This study
pBBR1-MCS3-*lacZ*-P4-98	Tc^r, Promoter 4(98 nt upstream) from *isc suf* operon on pBBR1-MCS3-*lacZ*	This study
pBBR1-MCS3-*lacZ*-P5-88	Tc^r, Promoter 5(88 nt upstream) from *isc suf* operon on pBBR1-MCS3-*lacZ*	This study
pBBR1-MCS3-*lacZ*-P5-112	Tc^r, Promoter 5(112 nt upstream) from *isc suf* operon on pBBR1-MCS3-*lacZ*	This study
pBBR1-MCS3-*lacZ*-P12	Tc^r, Promoter 1 and 2 from *isc suf* operon on pBBR1-MCS3-*lacZ*	This study
pBBR1-MCS3-*lacZ*-P25-88	Tc^r, Promoter 2 and 5 (88 nt upstream) from *isc suf* operon on pBBR1-MCS3-*lacZ*	This study
pBBR1-MCS3-*lacZ*-P25-112	Tc^r, Promoter 2 and 5 (112 nt upstream) from *isc suf* operon on pBBR1-MCS3-*lacZ*	This study
pBBR1-MCS3-*lacZ*-P125-88	Tc^r, Promoter 1, 2 and 5 (88 nt upstream) from *isc suf* operon on pBBR1-MCS3-*lacZ*	This study
pBBR1-MCS3-*lacZ*-P125-112	Tc^r, Promoter 1, 2 and 5 (112 nt upstream) from *isc suf* operon on pBBR1-MCS3-*lacZ*	This study
pBBR1-MCS3-*lacZ*-P254-60	Tc^r, Promoter 2, 5 and 4(60 nt upstream) from *isc suf* operon on pBBR1-MCS3-*lacZ*	This study
pBBR1-MCS3-*lacZ*-P254-98	Tc^r, Promoter 2, 5 and 4(98 nt upstream) from *isc suf* operon on pBBR1-MCS3-*lacZ*	This study
pBBR1-MCS3-*lacZ*-P1254-60	Tc^r, Promoter 1, 2, 5 and 4(60 nt upstream) from *isc suf* operon on pBBR1-MCS3-*lacZ*	This study
pBBR1-MCS3-*lacZ*-P12543	Tc^r, Promoter 1, 2, 5, 4 and 3 from *isc suf* operon on pBBR1-MCS3-*lacZ*	This study

pJET1.2-mut P5-88	Ap^r, mutated Promoter 5 (88 nt upstream) from *isc suf* operon on pJET1.2	This study
pJET1.2-mut P25-88	Ap^r, mutated Promoter 5 of P25 (88 nt upstream) from *isc suf* operon on pJET1.2	This study
pJET1.2-mut P4-60	Ap^r, mutated Promoter 4 (60 nt upstream) from *isc suf* operon on pJET1.2	This study
pJET1.2-mut P4-98	Ap^r, mutated Promoter 4 (98 nt upstream) from *isc suf* operon on pJET1.2	This study
pJET1.2-mut P254-60	Ap^r, mutated Promoter 4 of P254 (60 nt upstream) from *isc suf* operon on pJET1.2	This study
pJET1.2-mut P254-98	Ap^r, mutated Promoter 4 of P254 (98 nt upstream) from *isc suf* operon on pJET1.2	This study
pBBR1-MCS3-*lacZ*-mut P5-88	Tc^r, mutated Promoter 5 (88 nt upstream) from *isc suf* operon on pBBR1-MCS3-*lacZ*	This study
pBBR1-MCS3-*lacZ*-mut P25-88	Tc^r, mutated Promoter 5 of P25 (88 nt upstream) from *isc suf* operon on pBBR1-MCS3-*lacZ*	This study
pBBR1-MCS3-*lacZ*-mut P4-60	Tc^r, mutated Promoter 4 (60 nt upstream) from *isc suf* operon on pBBR1-MCS3-*lacZ*	This study
pBBR1-MCS3-*lacZ*-mut P4-98	Tc^r, mutated Promoter 4 (98 nt upstream) from *isc suf* operon on pBBR1-MCS3-*lacZ*	This study
pBBR1-MCS3-*lacZ*-mutP254-60	Tc^r, mutated Promoter 4 of P254 (60 nt upstream) from *isc suf* operon on pBBR1-MCS3-*lacZ*	This study
pBBR1-MCS3-*lacZ*-mutP254-98	Tc^r, mutated Promoter 4 of P254 (98 nt upstream) from *isc suf* operon on pBBR1-MCS3-*lacZ*	This study
pPHU281ΔRSP_3341	Tc^r, pPHU281 containing RSP_3341 gene with flanking sites	This study
pPHU281ΔRSP_3341::Sp	Tc^r, Sp^r,pPHU281ΔRSP_3341 containing Sp^r cassette	This study
pPHU281ΔRSP_2888	Tc^r, pPHU281 containing RSP_2888 gene with flanking sites	This study
pPHU281ΔRSP_2888::Km	Tc^r, Km^r, pPHU281ΔRSP_3341 containing Km^r cassette	This study
pPH45_Ω	Sp^r, source of Ω-Sp^r cassette	[13]
pPH45_Km	Km^r, source of Km^r cassette	[13]

Sp^r, spectinomycin-resistant; Ap^r, ampicillin-resistant; Tc^r, tetracycline-resistant; Km^r, kanamycin resistant; Gm^r, gentamicin resistant.

References

1. Rodionov, D. A.; Gelfand, M. S.; Todd, J. D.; Curson, A. R. J.; Johnston, A. W. B., Computational reconstruction of iron- and manganese-responsive transcriptional networks in alpha-proteobacteria. *Plos Computational Biology* **2006,** *2* (12), 1568-1585.
2. Peuser, V.; Remes, B.; Klug, G., Role of the Irr Protein in the Regulation of Iron Metabolism in Rhodobacter sphaeroides. *Plos One* **2012,** *7* (8).
3. van Niel, C. B., The Culture, General Physiology, Morphology, and Classification of the Non-Sulfur Purple and Brown Bacteria. *Bacteriological reviews* **1944,** *8* (1), 1-118.
4. Remes, B.; Eisenhardt, B. D.; Srinivasan, V.; Klug, G., IscR of *Rhodobacter sphaeroides* functions as repressor of genes for iron-sulfur metabolism and represents a new type of iron-sulfur-binding protein. *Microbiologyopen* **2015,** *4* (5), 790-802.
5. Peuser, V.; Metz, S.; Klug, G., Response of the photosynthetic bacterium *Rhodobacter sphaeroides* to iron limitation and the role of a Fur orthologue in this response. *Environmental microbiology reports* **2011,** *3* (3), 397-404.
6. Zeller, T.; Klug, G., Detoxification of hydrogen peroxide and expression of catalase genes in *Rhodobacter*. *Microbiology* **2004,** *150* (Pt 10), 3451-62.
7. Yanischperron, C.; Vieira, J.; Messing, J., Improved M13 Phage Cloning Vectors and Host Strains - Nucleotide-Sequences of the M13mp18 and Puc19 Vectors. *Gene* **1985,** *33* (1), 103-119.
8. Simon, R.; Oconnell, M.; Labes, M.; Puhler, A., Plasmid Vectors for the Genetic-Analysis and Manipulation of *Rhizobia* and Other Gram-Negative Bacteria. *Method Enzymol* **1986,** *118*, 640-659.
9. Zeller, T.; Mraheil, M. A.; Moskvin, O. V.; Li, K. Y.; Gomelsky, M.; Klug, G., Regulation of hydrogen peroxide-dependent gene expression in *Rhodobacter sphaeroides*: Regulatory functions of OxyR. *Journal of Bacteriology* **2007,** *189* (10), 3784-3792.
10. Hubner, P.; Willison, J. C.; Vignais, P. M.; Bickle, T. A., Expression of Regulatory Nif Genes in *Rhodobacter-Capsulatus*. *Journal of Bacteriology* **1991,** *173* (9), 2993-2999.
11. Mank, N. N.; Berghoff, B. A.; Hermanns, Y. N.; Klug, G., Regulation of bacterial photosynthesis genes by the small noncoding RNA PcrZ. *Proc Natl Acad Sci U S A* **2012,** *109* (40), 16306-11.
12. Kovach, M. E.; Elzer, P. H.; Hill, D. S.; Robertson, G. T.; Farris, M. A.; Roop, R. M., 2nd; Peterson, K. M., Four new derivatives of the broad-host-range cloning vector pBBR1MCS, carrying different antibiotic-resistance cassettes. *Gene* **1995,** *166* (1), 175-6.
13. Prentki, P.; Binda, A.; Epstein, A., Plasmid vectors for selecting IS1-promoted deletions in cloned DNA: sequence analysis of the omega interposon. *Gene* **1991,** *103* (1), 17-23.
